# 2-[(3-Bromo­anilino)meth­yl]-1,2-benzo­thia­zol-3(2*H*)-one

**DOI:** 10.1107/S1600536812012263

**Published:** 2012-04-04

**Authors:** Xiang-Hui Wang, Jian-Xin Yang, Qiang Lin, Jun-Hua Chen

**Affiliations:** a Institute of Environmental Science and Engineering, Kunming University of Science and Technology, Kunming 650093, People’s Republic of China; b Institute of Materials and Chemical Engineering, Hainan University, Haikou 570228, People’s Republic of China; c College of Chemistry and Chemical Engineering, Hainan Normal University, Haikou 571100, People’s Republic of China

## Abstract

The title compound, C_14_H_11_BrN_2_OS, was synthesized by the reaction of 1,2-benzothia­zol-3(2*H*)-one with formalin and 3-bromo­aniline in ethanol. The 1,2-benzothia­zolone ring system is approximately planar [maximum deviation = 0.0142 **(s.u.?)** Å] and forms a dihedral angle of 79.19 (5)° with the benzene ring. In the crystal, molecules are linked by N—H⋯O, C—H⋯O and C—H⋯Br interactions.

## Related literature

For background to the synthesis of benzoisothia­zolone deriv­atives, see: Davis (1972 ▶); Elgazwy & Abdel-Sattar (2003 ▶). For the biological activity of 1,2-benzoisothia­zolone derivatives, see: Taubert *et al.* (2002 ▶). For structural studies of related alkyl 3-oxo-2,3-dihydro-1,2-benzothia­zole-2-carboxyl­ate derivatives, see: Wang *et al.* (2011 ▶); Wang, Yang *et al.* (2011*a* ▶,*b* ▶)
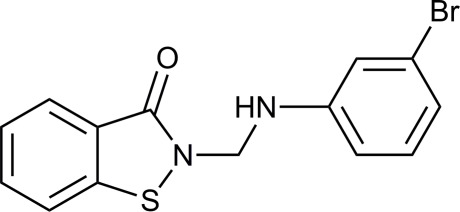


## Experimental

### 

#### Crystal data

C_14_H_11_BrN_2_OS
*M* *_r_* = 335.22
Monoclinic, 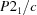

*a* = 7.351 (2) Å
*b* = 22.781 (7) Å
*c* = 8.559 (3) Å
β = 109.580 (4)°
*V* = 1350.5 (7) Å^3^
*Z* = 4
Mo *K*α radiation
μ = 3.19 mm^−1^
*T* = 153 K
0.38 × 0.37 × 0.31 mm

#### Data collection

Rigaku AFC10/Saturn724+ diffractometer
Absorption correction: multi-scan (*ABSCOR*; Higashi, 1995 ▶) *T*_min_ = 0.374, *T*_max_ = 0.436
11631 measured reflections
3587 independent reflections
2709 reflections with *I* > 2σ(*I*)
*R*_int_ = 0.037

#### Refinement

*R*[*F* ^2^ > 2σ(*F* ^2^)] = 0.032
*wR*(*F* ^2^) = 0.069
*S* = 1.00
3587 reflections
176 parameters
H atoms treated by a mixture of independent and constrained refinement
Δρ_max_ = 0.44 e Å^−3^
Δρ_min_ = −0.52 e Å^−3^

### 

Data collection: *CrystalClear* (Rigaku, 2008 ▶); cell refinement: *CrystalClear*; data reduction: *CrystalClear*; program(s) used to solve structure: *SHELXS97* (Sheldrick, 2008 ▶); program(s) used to refine structure: *SHELXL97* (Sheldrick, 2008 ▶); molecular graphics: *SHELXTL* (Sheldrick, 2008 ▶) and *DIAMOND* (Brandenburg, 1999 ▶); software used to prepare material for publication: *SHELXTL* and *publCIF* (Westrip, 2010 ▶).

## Supplementary Material

Crystal structure: contains datablock(s) I, global. DOI: 10.1107/S1600536812012263/rz2723sup1.cif

Structure factors: contains datablock(s) I. DOI: 10.1107/S1600536812012263/rz2723Isup2.hkl

Supplementary material file. DOI: 10.1107/S1600536812012263/rz2723Isup3.cml

Additional supplementary materials:  crystallographic information; 3D view; checkCIF report

## Figures and Tables

**Table 1:** Hydrogen-bond geometry (Å, °)

*D*—H⋯*A*	*D*—H	H⋯*A*	*D*⋯*A*	*D*—H⋯*A*
N2—H2*N*⋯O1^i^	0.82 (2)	2.14 (2)	2.939 (2)	167 (2)
C3—H3⋯O1^ii^	0.95	2.59	3.484 (3)	157
C10—H10⋯Br1^iii^	0.95	2.93	3.545 (3)	124
C14—H14⋯O1^i^	0.95	2.46	3.207 (3)	135

Symmetry codes: (i) 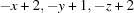; (ii) 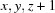; (iii) 
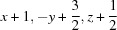
.

**Fig. 1.**
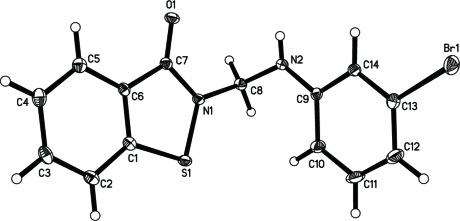
The molecular structure of the title compound with displacement ellipsoids drawn at the 30% probability level. H atoms are presented as small spheres of arbitrary radius.

**Fig. 2.**
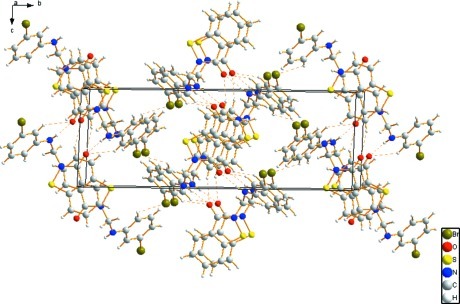
A view of the C—H···O and C—H···Br interactions (dashed lines) in the crystal structure of the title compound.

## supplementary crystallographic information

### Comment

The work presented herein is part of our interest in synthesizing various benzisothiazolone derivatives and confirming their structures by X-ray analysis (Wang *et al.*, 2011; Wang, Yang *et al.*, 2011*a*,*b*). These compounds will be utilized for the study of comparative bioactivity.

The molecular structure of the title compound is shown in Fig. 1. In the molecule, the benzisothiazolone ring system is approximately planar with a maximum deviation from the mean plane of 0.0142 A ° for the atom C1, and the dihedral angle between the benzene ring and the benzisothiazolone ring is 79.169 (5)°. In the crystal structure, intermolecular N—H···O, C—H···O and C—H···Br hydrogen bonds (Table 1) link molecules into a three-dimensional network (Fig. 2).

### Experimental

An ethanol solution (20 ml) containing 1,2-benzothiazol-3(2*H*)-one (1.51 g, 0.01 mol), formalin (1 mL) and 3-bromoaniline (1.72 g, 0.01 mol) was stirred at room temperature for 4.5 h to afford the title compound (1.97 g, yield 58.8%). Single crystals suitable for X-ray measurements were obtained by recrystallization of the title compound from trichloromethane/methanol (1:1 *v*/*v*) at room temperature.

### Refinement

The amine H atom was located in a difference Fourier map and refined freely. All other H atoms were placed at calculated positions and refined in riding mode, with C—H = 0.95–0.99 Å. The *U*_iso_(H) values were constrained to be 1.5*U*_eq_(C) for the methyl H atoms or 1.2*U*_eq_(C) for the remaining H atoms.

### Crystal data

**Table:**

C_14_H_11_BrN_2_OS	*F*(000) = 672
*M**_r_* = 335.22	*D*_x_ = 1.649 Mg m^−^^3^
Monoclinic, *P*2_1_/*c*	Mo *K*α radiation, λ = 0.71073 Å
Hall symbol: -P 2ybc	Cell parameters from 4184 reflections
*a* = 7.351 (2) Å	θ = 2.7–29.1°
*b* = 22.781 (7) Å	µ = 3.19 mm^−^^1^
*c* = 8.559 (3) Å	*T* = 153 K
β = 109.580 (4)°	Block, colourless
*V* = 1350.5 (7) Å^3^	0.38 × 0.37 × 0.31 mm
*Z* = 4	

### Data collection

**Table:**

Rigaku AFC10/Saturn724+ diffractometer	3587 independent reflections
Radiation source: Rotating Anode	2709 reflections with *I* > 2σ(*I*)
Graphite monochromator	*R*_int_ = 0.037
Detector resolution: 28.5714 pixels mm^-1^	θ_max_ = 29.1°, θ_min_ = 2.7°
phi and ω scans	*h* = −10→9
Absorption correction: multi-scan (*ABSCOR*; Higashi, 1995)	*k* = −31→30
*T*_min_ = 0.374, *T*_max_ = 0.436	*l* = −11→11
11631 measured reflections	

### Refinement

**Table:**

Refinement on *F*^2^	Primary atom site location: structure-invariant direct methods
Least-squares matrix: full	Secondary atom site location: difference Fourier map
*R*[*F*^2^ > 2σ(*F*^2^)] = 0.032	Hydrogen site location: inferred from neighbouring sites
*wR*(*F*^2^) = 0.069	H atoms treated by a mixture of independent and constrained refinement
*S* = 1.00	*w* = 1/[σ^2^(*F*_o_^2^) + (0.0289*P*)^2^ + 0.160*P*] where *P* = (*F*_o_^2^ + 2*F*_c_^2^)/3
3587 reflections	(Δ/σ)_max_ < 0.001
176 parameters	Δρ_max_ = 0.44 e Å^−^^3^
0 restraints	Δρ_min_ = −0.52 e Å^−^^3^

### Special details

**Table:**

Geometry. All e.s.d.'s (except the e.s.d. in the dihedral angle between two l.s. planes) are estimated using the full covariance matrix. The cell e.s.d.'s are taken into account individually in the estimation of e.s.d.'s in distances, angles and torsion angles; correlations between e.s.d.'s in cell parameters are only used when they are defined by crystal symmetry. An approximate (isotropic) treatment of cell e.s.d.'s is used for estimating e.s.d.'s involving l.s. planes.
Refinement. Refinement of *F*^2^ against ALL reflections. The weighted *R*-factor *wR* and goodness of fit *S* are based on *F*^2^, conventional *R*-factors *R* are based on *F*, with *F* set to zero for negative *F*^2^. The threshold expression of *F*^2^ > σ(*F*^2^) is used only for calculating *R*-factors(gt) *etc*. and is not relevant to the choice of reflections for refinement. *R*-factors based on *F*^2^ are statistically about twice as large as those based on *F*, and *R*- factors based on ALL data will be even larger.

### Fractional atomic coordinates and isotropic or equivalent isotropic displacement parameters (Å^2^)

**Table:**

	*x*	*y*	*z*	*U*_iso_*/*U*_eq_	
Br1	0.37553 (3)	0.693276 (11)	0.83530 (3)	0.03101 (8)	
O1	1.1959 (2)	0.47887 (6)	1.16801 (16)	0.0223 (3)	
S1	1.30876 (8)	0.60263 (2)	1.48237 (6)	0.02068 (12)	
N1	1.2660 (2)	0.56804 (7)	1.29651 (19)	0.0181 (4)	
C13	0.6410 (3)	0.69583 (9)	0.9710 (3)	0.0220 (4)	
C7	1.2302 (3)	0.50892 (9)	1.2939 (2)	0.0174 (4)	
N2	1.0771 (2)	0.60956 (8)	1.0275 (2)	0.0184 (4)	
C5	1.2107 (3)	0.43397 (10)	1.5099 (3)	0.0218 (4)	
H5	1.1821	0.4025	1.4327	0.026*	
C3	1.2648 (3)	0.47107 (10)	1.7852 (3)	0.0253 (5)	
H3	1.2716	0.4638	1.8963	0.030*	
C1	1.2822 (3)	0.53648 (9)	1.5741 (2)	0.0181 (4)	
C9	0.9559 (3)	0.65290 (9)	1.0506 (2)	0.0172 (4)	
C10	1.0203 (3)	0.69745 (9)	1.1676 (3)	0.0261 (5)	
H10	1.1519	0.6985	1.2368	0.031*	
C14	0.7614 (3)	0.65249 (9)	0.9498 (2)	0.0182 (4)	
H14	0.7134	0.6229	0.8682	0.022*	
C6	1.2401 (3)	0.49047 (9)	1.4604 (2)	0.0168 (4)	
C8	1.2648 (3)	0.59860 (9)	1.1440 (2)	0.0184 (4)	
H8A	1.3328	0.6366	1.1755	0.022*	
H8B	1.3384	0.5747	1.0891	0.022*	
C2	1.2955 (3)	0.52715 (10)	1.7396 (3)	0.0237 (5)	
H2	1.3246	0.5584	1.8174	0.028*	
C4	1.2239 (3)	0.42447 (10)	1.6723 (3)	0.0259 (5)	
H4	1.2051	0.3861	1.7078	0.031*	
C12	0.7015 (3)	0.74034 (11)	1.0860 (3)	0.0324 (5)	
H12	0.6149	0.7697	1.0973	0.039*	
C11	0.8935 (4)	0.74030 (11)	1.1840 (3)	0.0349 (6)	
H11	0.9402	0.7703	1.2645	0.042*	
H2N	1.017 (3)	0.5816 (10)	0.975 (3)	0.023 (6)*	

### Atomic displacement parameters (Å^2^)

**Table:**

	*U*^11^	*U*^22^	*U*^33^	*U*^12^	*U*^13^	*U*^23^
Br1	0.02204 (12)	0.03134 (14)	0.03771 (15)	0.00613 (10)	0.00744 (9)	0.00158 (11)
O1	0.0263 (8)	0.0235 (8)	0.0165 (8)	−0.0016 (7)	0.0064 (6)	−0.0054 (6)
S1	0.0272 (3)	0.0200 (3)	0.0155 (3)	−0.0026 (2)	0.0080 (2)	−0.0035 (2)
N1	0.0253 (9)	0.0177 (9)	0.0124 (9)	−0.0007 (7)	0.0076 (7)	−0.0009 (7)
C13	0.0227 (10)	0.0203 (11)	0.0235 (12)	0.0014 (9)	0.0083 (9)	0.0035 (9)
C7	0.0150 (9)	0.0194 (11)	0.0166 (11)	0.0010 (8)	0.0038 (8)	−0.0013 (8)
N2	0.0189 (9)	0.0179 (9)	0.0162 (9)	0.0000 (7)	0.0029 (7)	−0.0024 (7)
C5	0.0182 (10)	0.0234 (11)	0.0229 (12)	0.0000 (9)	0.0059 (8)	0.0005 (9)
C3	0.0220 (11)	0.0365 (13)	0.0191 (12)	0.0016 (10)	0.0092 (9)	0.0068 (10)
C1	0.0137 (9)	0.0231 (11)	0.0178 (11)	−0.0009 (8)	0.0054 (8)	0.0001 (8)
C9	0.0207 (10)	0.0151 (10)	0.0164 (10)	0.0000 (8)	0.0071 (8)	0.0024 (8)
C10	0.0239 (11)	0.0211 (11)	0.0289 (13)	−0.0026 (10)	0.0032 (9)	−0.0066 (9)
C14	0.0228 (11)	0.0176 (10)	0.0150 (11)	−0.0001 (8)	0.0073 (8)	0.0006 (8)
C6	0.0128 (9)	0.0212 (10)	0.0155 (10)	0.0019 (8)	0.0038 (7)	0.0014 (8)
C8	0.0208 (10)	0.0215 (11)	0.0154 (10)	−0.0010 (9)	0.0095 (8)	0.0005 (8)
C2	0.0229 (11)	0.0314 (13)	0.0177 (11)	0.0002 (9)	0.0079 (9)	−0.0016 (9)
C4	0.0232 (11)	0.0277 (12)	0.0279 (13)	0.0004 (10)	0.0098 (9)	0.0081 (10)
C12	0.0337 (13)	0.0256 (12)	0.0389 (14)	0.0061 (11)	0.0134 (11)	−0.0091 (11)
C11	0.0392 (14)	0.0251 (13)	0.0382 (14)	−0.0003 (11)	0.0100 (11)	−0.0165 (11)

### Geometric parameters (Å, º)

**Table:**

Br1—C13	1.908 (2)	C3—C4	1.398 (3)
O1—C7	1.229 (2)	C3—H3	0.9500
S1—N1	1.7074 (17)	C1—C6	1.393 (3)
S1—C1	1.740 (2)	C1—C2	1.403 (3)
N1—C7	1.371 (3)	C9—C10	1.393 (3)
N1—C8	1.476 (2)	C9—C14	1.401 (3)
C13—C14	1.378 (3)	C10—C11	1.389 (3)
C13—C12	1.379 (3)	C10—H10	0.9500
C7—C6	1.464 (3)	C14—H14	0.9500
N2—C9	1.388 (3)	C8—H8A	0.9900
N2—C8	1.427 (2)	C8—H8B	0.9900
N2—H2N	0.82 (2)	C2—H2	0.9500
C5—C4	1.378 (3)	C4—H4	0.9500
C5—C6	1.394 (3)	C12—C11	1.379 (3)
C5—H5	0.9500	C12—H12	0.9500
C3—C2	1.376 (3)	C11—H11	0.9500
			
N1—S1—C1	90.41 (9)	C11—C10—H10	119.8
C7—N1—C8	120.30 (16)	C9—C10—H10	119.8
C7—N1—S1	116.28 (13)	C13—C14—C9	118.83 (19)
C8—N1—S1	123.42 (13)	C13—C14—H14	120.6
C14—C13—C12	123.6 (2)	C9—C14—H14	120.6
C14—C13—Br1	117.97 (16)	C1—C6—C5	120.22 (18)
C12—C13—Br1	118.46 (17)	C1—C6—C7	113.01 (18)
O1—C7—N1	122.90 (18)	C5—C6—C7	126.76 (18)
O1—C7—C6	128.46 (19)	N2—C8—N1	114.72 (16)
N1—C7—C6	108.64 (17)	N2—C8—H8A	108.6
C9—N2—C8	122.78 (18)	N1—C8—H8A	108.6
C9—N2—H2N	112.0 (16)	N2—C8—H8B	108.6
C8—N2—H2N	117.8 (16)	N1—C8—H8B	108.6
C4—C5—C6	119.1 (2)	H8A—C8—H8B	107.6
C4—C5—H5	120.5	C3—C2—C1	117.7 (2)
C6—C5—H5	120.5	C3—C2—H2	121.2
C2—C3—C4	121.8 (2)	C1—C2—H2	121.2
C2—C3—H3	119.1	C5—C4—C3	120.3 (2)
C4—C3—H3	119.1	C5—C4—H4	119.9
C6—C1—C2	121.0 (2)	C3—C4—H4	119.9
C6—C1—S1	111.65 (15)	C13—C12—C11	116.9 (2)
C2—C1—S1	127.33 (17)	C13—C12—H12	121.5
N2—C9—C10	122.76 (19)	C11—C12—H12	121.5
N2—C9—C14	118.63 (19)	C12—C11—C10	121.6 (2)
C10—C9—C14	118.60 (19)	C12—C11—H11	119.2
C11—C10—C9	120.5 (2)	C10—C11—H11	119.2
			
C1—S1—N1—C7	0.55 (16)	S1—C1—C6—C7	0.5 (2)
C1—S1—N1—C8	−179.97 (16)	C4—C5—C6—C1	0.1 (3)
C8—N1—C7—O1	0.0 (3)	C4—C5—C6—C7	−179.39 (19)
S1—N1—C7—O1	179.45 (15)	O1—C7—C6—C1	−179.89 (19)
C8—N1—C7—C6	−179.87 (16)	N1—C7—C6—C1	−0.1 (2)
S1—N1—C7—C6	−0.4 (2)	O1—C7—C6—C5	−0.4 (3)
N1—S1—C1—C6	−0.57 (15)	N1—C7—C6—C5	179.41 (18)
N1—S1—C1—C2	−179.34 (19)	C9—N2—C8—N1	74.6 (2)
C8—N2—C9—C10	14.7 (3)	C7—N1—C8—N2	76.3 (2)
C8—N2—C9—C14	−166.54 (18)	S1—N1—C8—N2	−103.16 (19)
N2—C9—C10—C11	179.0 (2)	C4—C3—C2—C1	0.7 (3)
C14—C9—C10—C11	0.3 (3)	C6—C1—C2—C3	−0.2 (3)
C12—C13—C14—C9	0.5 (3)	S1—C1—C2—C3	178.47 (16)
Br1—C13—C14—C9	−178.70 (15)	C6—C5—C4—C3	0.4 (3)
N2—C9—C14—C13	−179.28 (18)	C2—C3—C4—C5	−0.9 (3)
C10—C9—C14—C13	−0.5 (3)	C14—C13—C12—C11	−0.3 (4)
C2—C1—C6—C5	−0.2 (3)	Br1—C13—C12—C11	178.94 (18)
S1—C1—C6—C5	−179.05 (15)	C13—C12—C11—C10	0.0 (4)
C2—C1—C6—C7	179.34 (18)	C9—C10—C11—C12	0.0 (4)

### Hydrogen-bond geometry (Å, º)

**Table:**

*D*—H···*A*	*D*—H	H···*A*	*D*···*A*	*D*—H···*A*
N2—H2*N*···O1^i^	0.82 (2)	2.14 (2)	2.939 (2)	167 (2)
C3—H3···O1^ii^	0.95	2.59	3.484 (3)	157
C10—H10···Br1^iii^	0.95	2.93	3.545 (3)	124
C14—H14···O1^i^	0.95	2.46	3.207 (3)	135

Symmetry codes: (i) −*x*+2, −*y*+1, −*z*+2; (ii) *x*, *y*, *z*+1; (iii) *x*+1, −*y*+3/2, *z*+1/2.
